# Acute Myocardial Infarction During the Last Part of a Triathlon: A Case Report

**DOI:** 10.7759/cureus.32768

**Published:** 2022-12-21

**Authors:** Satoshi Yamaguchi, Michio Shimabukuro

**Affiliations:** 1 Department of Diabetes, Endocrinology and Metabolism, School of Medicine, Fukushima Medical University, Fukushima, JPN; 2 Department of Cardiology, Nakagami Hospital, Okinawa, JPN

**Keywords:** ventricular fibrillation, resuscitation, sudden cardiac death, acute myocardial infarction, triathlon

## Abstract

Triathlon has a risk of sudden cardiac death (SCD) for athletes. Most SCDs during endurance sports in adult athletes are due to coronary artery disease but often lack signs during working out. Here, we report the acute myocardial infarction subsequent to the plaque rapture while running, the last part of a triathlon. A 44-year-old Asian athlete hardly worked out for the triathlon. An annual medical checkup did not reveal any abnormalities. He experienced ventricular fibrillation while running, the last part of a triathlon. The other athletes provided immediate cardiopulmonary resuscitation. Coronary artery angiography revealed acute myocardial infarction in the left anterior descending artery. Stenting was performed. He was discharged without any neurological complications on the 14th day of hospitalization. Athletes should be aware of the risk of SCD during endurance sports and educate themselves to provide basic life support when they encounter other athlete suffering from sudden cardiac attacks.

## Introduction

Sudden cardiac death (SCD) can occur during marathons and triathlons, although it is extremely rare, even in adult athletes who train regularly [1]. SCD in endurance sports is due to arrhythmia, cardiomyopathy, congenital heart disease, and a variety of other causes, but coronary artery disease is the most common cause in over-35-year-old athletes [2]. Screening for chest symptoms and family history is recommended [3]. Considering the low incidence of SCD [4], it is impractical to identify true high-risk athletes. Moreover, athletes cannot participate in competitions if they are identified as high risk at screening. Thus, a one-size-fits-all screening may not always be useful. We can learn important lessons in risk management for athletic competitions by reviewing life-saving cases of cardiac arrest during sports with a good neurological prognosis. Herein, we report a case of cardiac arrest during a triathlon in a 44-year-old Asian athlete.

## Case presentation

A 44-year-old Asian man had been training himself for three years for participation in triathlons and had completed seven of the eight attempts. For his training, he was on a strict low-carbohydrate diet; he did not eat rice but mainly consumed lean meat. He had never smoked. He was regularly swimming one to two kilometers and worked out on his own. The patient had never experienced chest pain during his workouts. The patient’s father had a history of coronary disease and had undergone coronary artery bypass surgery 20 years back. Neither dyslipidemia, such as high low-density lipoprotein cholesterol, nor abnormal electrocardiogram findings were found in his annual medical checkup.

**Figure 1:**
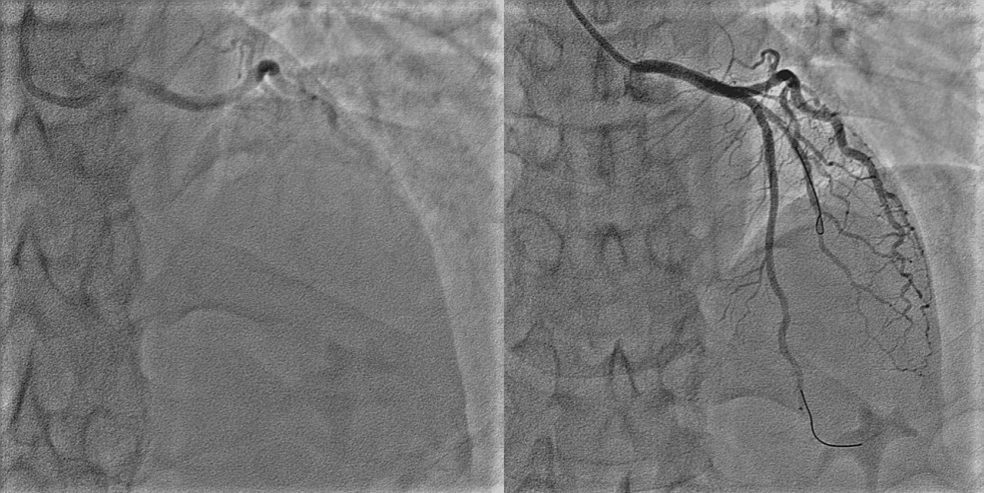
Coronary angiography (Left) Coronary angiography showed that the left anterior descending artery was occluded by acute thrombosis. (Right) Blood flow in the left descending artery is reestablished after thrombus aspiration and stenting.

During the triathlon, the patient completed swimming and cycling, without any problems. He suddenly collapsed during the running part, which is usually the last part of a triathlon. His fellow participants received bystander cardiopulmonary resuscitation. The emergency medical team detected ventricular fibrillation; therefore, the patient was shocked twice using an automated electronic defibrillator. The patient's spontaneous circulation returned while running. The patient was then transported to the hospital. His Glasgow Coma Scale score was E4V4M6. Electrocardiography showed ST-T segment elevation at the V1-V4 leads. Coronary angiography revealed occlusion in the proximal left descending artery due to plaque rupture and acute thrombosis (Figure 1).

**Figure 2:**
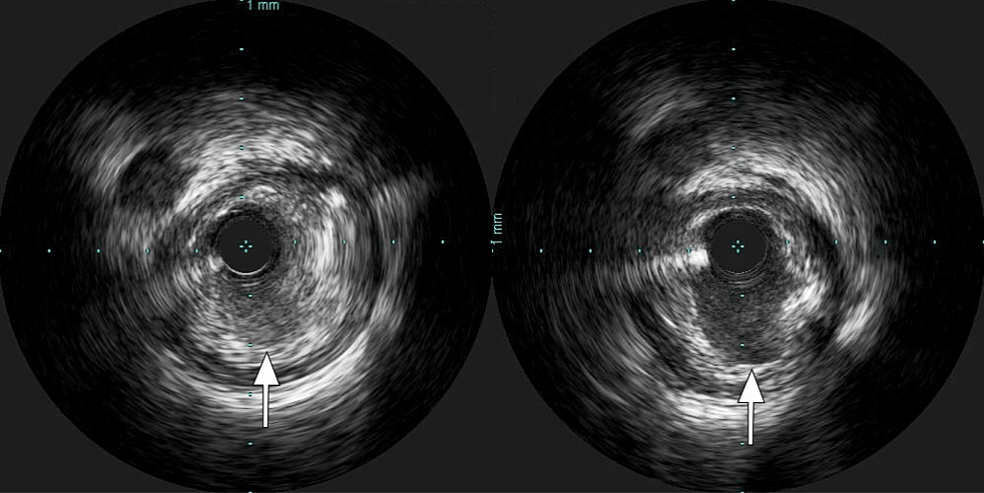
Intravascular ultrasound sonography (Left) Plaque rupture and thrombosis around low echoic plaque in intravascular ultrasonography. The white arrow indicates raptured plaque filled with thrombus. (Right) The white arrow indicates an ulcer discovered after thrombosis aspiration.

Intravascular ultrasonography revealed low echoic plaque richness in the left anterior descending coronary artery and plaque rupture in the culprit artery (Figure 2). Thrombectomy and direct stenting were then performed. The patient's blood flow recovered to the level of thrombolysis in myocardial infarction grade 3. His creatinine phosphokinase level peaked at 3534 IU/L and creatinine phosphokinase-MB peaked at 479 IU/L. Laboratory tests revealed a triglyceride level of 77 mg/dL, a low-density cholesterol level of 100 mg/dL, and a high-density cholesterol level of 43 mg/dL on admission.

The patient's hospital stay was uneventful, and he was discharged on the 14th hospital day without any neurological complications.

Written informed consent for publication of this case was obtained from the patient.

## Discussion

This case presents an acute myocardial infarction that occurred in the last part of a triathlon. This case report presents two important findings. Firstly, this is a case of an athlete without any symptoms, who experienced sudden cardiac arrest while running, the last part of the triathlon. Second, a bystander on-road triathlete, not a first-aid station or emergency medical service, could provide basic life support immediately.

A large number of retrospective observational studies [5] reported that 14 of 959214 triathlon participants died. Of these patients, 13 died while swimming, and the remaining one died during cycling [5]. An autopsy revealed that aesthetes who died during swimming or biking had cardiovascular diseases, such as left ventricular hypertrophy and congenital coronary artery abnormalities [5]. In contrast, our patient did not have any structural heart diseases. Notably, ventricular fibrillation subsequent to acute myocardial infarction occurs during running as the last part of the triathlon. In our case, the patient did not have any symptoms during exercise and completed the swimming and cycling parts of the triathlon without any symptoms. Thus, the patient did not appear to have organic stenosis. Dehydration and rigorous workout may provoke plaque rupture and platelet activation, resulting in thrombosis formation around the vulnerable plaque [6]. We believe that this vulnerable plaque ruptured during the running section of the triathlon.

The European Society of Cardiology states that screening should focus on athletes at high risk of atherosclerotic cardiovascular disease prior to endurance sports [3]. They recommend that atherosclerotic cardiovascular disease risk should be estimated using the Systematic Coronary Risk Evaluation (SCORE) [3]. Additional examinations, including coronary artery calcification score, exercise stress echocardiography, and drug stress scintigraphy, may improve the diagnostic performance of chronic coronary artery disease [3]. Nevertheless, the American Heart Association does not recommend universal electrocardiography because of its feasibility, cost, quality assurance, and potential impact on an athlete's career [7]. Nevertheless, SCORE would determine a low risk in this patient. Therefore, for myocardial infarction caused by plaque rupture, scoring systems, and stress testing may provide little benefit. Tanaka et al. reported the effectiveness of a comprehensive approach for preventing SCD during marathons [8]. The comprehensive approach consisted of a running physician, roadside volunteers, and first aid stations providing basic life support, delivery of automated electrical discharge by mobile bicycle, mobile on-foot, activation of emergency medical services, and an information-sharing system [8]. They reported that such interventions treated 42 cardiac arrests, resulting in 39 cases with no or minor neurological complications [8]. To prevent cardiac death in athletes during endurance events such as marathons and triathlons, the standard medical screening prior to the event such as electrocardiogram and echocardiography cannot be enough to detect the risk of such acute myocardial infarction subsequent to the plaque rapture, but providing a comprehensive intervention for SCD is considered indispensable to save athletes from SCD.

## Conclusions

Comprehensive SCD prevention approaches that provide adequate basic life support, delivery of automated electronic defibrillators, and a call for emergency medical services may be more practical and effective than pre-competition screening.
